# A newly discovered Lnc-PDZD7-3 increased metastatic and proliferative potential of lung adenocarcinoma cells via modulating FN1/fibronectin signaling 

**DOI:** 10.3389/fgene.2025.1618449

**Published:** 2025-10-23

**Authors:** Guodong Zhang, Jianbo Zhang, Fachang Yu, Xiaohe Hao

**Affiliations:** ^1^ Thoracic Surgery Department, Shandong Cancer Hospital and Institute, Shandong First Medical University and Shandong Academy of Medical Sciences, Jinan, Shandong, China; ^2^ Department of Pathology, Shandong Cancer Hospital and Institute, Shandong First Medical University and Shandong Academy of Medical Sciences, Jinan, Shandong, China; ^3^ Department of Oncology, The Fifth People’s Hospital of Jinan, Jinan, Shandong, China; ^4^ Department of Clinical Laboratory, Shandong Cancer Hospital and Institute, Shandong First Medical University and Shandong Academy of Medical Sciences, Jinan, Shandong, China

**Keywords:** Lnc-PDZD7-3, lung adenocarcinoma, migration, proliferation, FN1

## Abstract

The global burden of lung adenocarcinoma (LUAD) has been on the rise, making it among the leading contributor to cancer-related deaths. Long non-coding RNA (lncRNA) are implicated in the initiation and progression of LUAD. To date, the mechanism by which lncRNA participate in LUAD are not clearly characterized. Here, we investigated the role of the newly-discovered Lnc-PDZD7-3 in the development of LUAD. Results revealed downregulation of Lnc-PDZD7-3 in human normal lung tissues and upregulation in LUAD tissues from the TCGA (The Cancer Genome Atlas) databases. Excessive expression of Lnc-PDZD7-3 promotes occurrence of distant metastasis. Lnc-PDZD7-3 knockdown suppressed the proliferative and viability potential of cells, as well enhanced apoptosis and inhibited the migratory activity of LUAD cells. Notably, expression levels of MMP9, Vimentin, Twist, Fibronectin, and MMP2 in LUAD cells were downregulated markedly except for snail following Lnc-PDZD7-3 knockdown. Through rescue experiments, we confirmed that Lnc-PDZD7-3 enhanced LUAD development by activating FN1/fibronectin signaling. Meanwhile, we also identified that Lnc-PDZD7-3 was localized in cytoplasm and nucleus segments of LUAD cells by FISH technology. In summary, this study implicates Lnc-PDZD7-3 in the pathomechanisms of LUAD via the FN1/fibronectin signaling, suggesting it may be diagnostic biomarker and therapeutic targets of LUAD.

## Introduction

Globally, over 2.2 million new cases of lung cancer are reported with about 1.9 million deaths due to cancer documented in 2020. For this reason, lung cancer is among the most common malignant cancers worldwide (Sung et al., 2021). There are two types of lung cancer: small cell and non-small cell. It is estimated that NSCLCs account for 80–85% of lung cancers such as, large-cell carcinoma, squamous cell carcinoma, and adenocarcinoma (Zappa and Mousa, 2016). Currently, the most frequent histological type of NSCLC is LUAD (Beasley et al., 2005). Approximately 40% of LUAD cases experience metastasis following the first diagnosis, and this leads to poor long-term clinical outcomes (Tamura et al., 2015). Epigenetic processes refers to modifications which are different from alterations in DNA sequences (Berger et al., 2009). LncRNA is a non-coding RNA that is more than 200 nucleotides in length, which does not code for specific proteins (Tang et al., 2018). Previous studies indicate that LncRNAs are important factors contributing to cancer development including LUAD (Fatemi et al., 2014; Li et al., 2020; Sun et al., 2017; Wu et al., 2017). Currently, the mechanisms driving LUAD development are not fully known.

Lnc-PDZD7-3 (Gene ID is ENSG00000273162.1 and transcript ID is ENST00000609242.1) was a recently identified lncRNA that modulates LUAD development. Lnc-PDZD7-3 is located on chromosome 10q24.31 and has a length of 977bps with 1 exon and associated with 245 variant alleles and mapped to 110 oligonucleotide probes. Nevertheless, little is known regarding the functions, expression profile, and clinical value of Lnc-PDZD7-3 in LUAD. Here, the Lnc-PDZD7-3 expression profile in LUAD tissue and cells was explored through analysis of gene profiling data from the TCGA (The Cancer Genome Atlas) database. The results were validated in a cohort of 57 paired tissues. Results demonstrated that Lnc-PDZD7-3 were higher in LUAD tissues than in normal lung tissues, and this upregulation was linked to distant metastases. We also found that Lnc-PDZD7-3 promoted the proliferative and metastatic potential of LUAD cells through a mechanism involving high expression of FN1 gene.

## Materials and methods

### Bioinformatics analysis

There are 585 LUAD samples with complete data were obtained from the TCGA database, comprising 57 samples of RNAseq data of paired tissue (cancerous and paracancerous tissues) and 514 samples with their corresponding pathological and RNAseq data.

### Cell selection and culture

The human LUAD cell lines H1975, 95D, A549, and H1299 were bought from the American Type Culture Collection (ATCC, Manassas, VA, United States). The A549 cells were cultured in F12K medium (ATCC, Manassas, VA) whereas the, other cell lines were cultured in the 1,640 medium (Corning Incorporated, Corning, NY, United States) containing with 10% fetal bovine serum (FBS) (Gibco, Thermo Fisher Scientific, Waltham, MA, United States) under humidified conditions at 37 °C with 5% CO_2_. At about 80–90% confluency, the cells were digested with trypsin (Sigma, St. Louis, Missouri) and subcultured or frozen for storage. Those showing good growth condition were chosen and subjected to analysis to determine the expression profile of lncRNA. H1975 cells, which exhibit altered IncRNA expression relative to normal lung cells and A549 cells with moderate differences were used for cell transfection and other experiments.

### Construction of RNA interference lentiviral vector and plasmid extraction

The following sequences of Lnc-PDZD7-3 were selected as interference targets: 5′-AGG​CCT​GGA​GCA​GAT​ATT​CAA-3'. Design and synthesize RNAi sequence: Single-stranded DNA oligo of sh-lncRNA (Table 1) was dissolved in annealing buffer (final concentration of 20 µM), then cooled naturally at 90 °C for 15 min to form double-stranded DNA oligo with sticky ends. A 20 μL reaction system was established in line with the manual guidelines on the Fermentas T4 DNA Ligase kit (Thermo Fisher Scientific, Inc.). The vector was constructed by ligating double-stranded DNA oligo with AgeI and EcoRI double digested linearized GV493. The ligation products were converted to competent *E. coli* cells, and the colonies were characterized as correct clones by PCR amplification. Positive clone sequencing was performed with the identification primers -F (5′-GGa​aag​aat​agt​aga​cat​A-3'), and the correctly sequenced bacteria were transferred into 150 mL LB liquid medium enriched with Amp antibiotics and incubated overnight at 37 °C with shaking. The Plasmid was extracted following to protocols of the EndoFree Maxi Plasmid Kit. The plasmid with qualified quality inspection was packaged with lentivirus by Shanghai Jikegenin Chemical Technology Co., LTD.

**TABLE 1:** Design and synthesize RNAi sequence: single-stranded DNA oligo of sh-lncRNA.

Internal number	5′additional base	STEM	Loop	STEM	3′additional base
psc71417-1	CCGG	AGG​CCT​GGA​GCA​GAT​ATT​CAA	CTCGAG	TTG​AAT​ATC​TGC​TCC​AGG​CCT	TTTTTG
psc71417-2	AATTCAAAAA	AGG​CCT​GGA​GCA​GAT​ATT​CAA	CTCGAG	TTG​AAT​ATC​TGC​TCC​AGG​CCT	

### Cell grouping and processing

A549 and H1975 cells in the logarithmic growth phase were transfected with lentivirus and divided into: Control group (NC) (cells infected with negative control virus), Knockdown group (shLncPDZD7-3 group, Lnc-PDZD7-3 gene interference virus and cells infected with negative control virus), OE group (Lnc-PDZD7-3 gene interference virus and downstream gene overexpression virus infected cell group). Downstream genes including TWIST1 FN1, SNAI2, VIM, MMP2, MMP9, produced by Shanghai JiKaiJi for chemical technology co., LTD.), and cell transfection efficiency about 80% normal is used to downstream experiment.

### Real time PCR

Total RNA samples were obtained from H1975 and A549 cells by treatment with the Trizol reagent (Invitrogen, Carlsbad, CA, United States). The extracted RNA was used to synthesize cDNA which was then subjected to PCR analysis using SYBR Master Mixture (Takara, Dalian, China). All primers utilized in this experiment were prepared by Guangzhou RiboBio Co., Ltd. The GAPDH mRNA served as the house-keeping gene. The PCR conditions were as follows: pre-denaturation (94 °C) for 5 min, denaturation (94 °C) for 40 s, annealing at 60 °C for 40 s, and DNA strand extension at 72 °C for 1 min. The reactions were run for a total of 40 cycles. The extension was performed at 72 °C for 10 min qRT-PCR was runs on a LightCycler 480 II real-time PCR system. The relative gene expression of the target gene was normalized to that of the control gene, and was calculated with the 2^−ΔΔCT^. ΔCt = Ct value of target gene - Ct value of reference gene; -ΔΔCt = Mean value of ΔCt in NC group -Mean value of ΔCt in experimental group.

### Western blotting

The concentration of protein samples extracted after transfection were determined with the BCA Protein Detection Kit (Thermo Fisher Scientific, Inc.). The samples were heated at 95 °C for 10 min for denaturation and then resolved on 10% SDS-PAGE under standard procedures. They were then transferred to polyvinylidene difluoride membranes (PVDF) (Millipore Corp., Bedford, Massachusetts), which was blocked with 5% fat-free milk for 1 h at room temperature, followed by an overnight incubation with the following primary antibodies at 4 °C: Twist (ab50887,abcam,1: 100), Fibronectin (MAB 1918, R&D,1:500), Snail (#3879,CST, 1:2000), Vimentin (#3932, CST,1:1000), MMP9(#13667, CST,1:500), MMP2(#40994, CST,1:500) and GAPDH (ab37168, abcam,1: 10,000). After washing thrice with TBST, the membranes were incubated with the respective secondary antibody conjugated to HRP for 30 min at room temperature, and washed four times with TBST on a shaker at room temperature for 5 min each. The protein bands were detected using the enhanced chemiluminescence (ECL) assay kit (ASPEN) and analyzed with the AlphaEaseFC software processing system.

### Celigo cell count

The transfected target cells were plated according to the growth rate. Each well of the culture system contained 100 μL of cell medium and equal number of cells were added to each well. The cells were cultured at 37 °C in 5%CO_2_ incubator and analyzed using the Celigo cell imaging analyzer once a day from the second day of culture until day 5. The ratio of the cell count at each time point of each group to the cell count value at the first day was calculated to obtain the proliferation multiple of the cells at each time point. Based on the time point and proliferation multiple, we plotted a growth curve in line with the cell proliferation multiple.

### Flow cytometry analyses

The transfected target cells were obtained in the same 5 mL centrifuge tube, and the cells were washed and precipitated once with D-Hanks and 1×binding buffer precooled at 4 °C. The cell suspension was centrifuged at 1300 rmp and 3 min to collect the cells. The cell precipitates were resuspended with 200 μL 1×binding buffer, then stained with 10 μL Annexin V-APC and kept out of light for 10–15 min at room temperature. The percentage of apoptotic cells was calculated and analyzed by BD C6 PLUS flow cytometry.

### Cell viability analysis and colony formation assay

Adding 20 μL of 5 mg/mL 3-(4, 5-Dimethyl-2-Thiazolyl)-2,5-diphenyl-2-H-tetrazolium bromide (MTT) to the microplate, after incubating with transfected cells in microwells for 4 h, all the culture medium was aspirated, and 100 μL Dimethyl sulfoxide (DMSO) was added to dissolve Azan granules. The oscillator was oscillated for 2–5 min, and the OD value was detected by microplate reader at 490/570 nm, and the viability of the cells were compared among the groups. The transfected cells of each group were spread on a 6-well plate and cultured continuously in an incubator for 14 days. The formation of cell clones was determined by fluorescence microscopy, stained with crystal violet and photographed for clone counting.

### Invasion chamber

Following the instruction manual of the Corning invasion kit, cells in serum-free medium were planted in Matrigel stromal layer after hydration with 1 × 10^5^ cells per well. 750 μL 30% FBS medium was added to the outer chamber and cultured in 37 °C incubator for 30 h. The cells were turned upside down on absorbent paper with the aim of discarding the culture medium, and the cells were removed by cotton swab. After staining the transferred cells for 3–5 min, the cells were rinsed and dried, and the field of vision was randomly selected in each cell for microscopical photography, and differences in cell invasion abilities between the groups were analyzed.

### Transwell assay

Cells from the serum-free medium were seeded at a density of 1 × 10^5^ cells per well in the upper chamber of the Transwell insert (Corning Incorporated, Corning, NY, United States). Subsequently, 600 µL of the medium containing 20% FBS was added to the lower chamber. The cells were incubated for 24 h at 37 °C the migrated cells to the lower surface were fixed in 4% formaldehyde (Solarbio Science&Technology Co., Beijing, China) and stained with 0.1% crystal violet (Solarbio Science&Technology Co.). Images were captured using an inverted microscope (XDS-100, Shanghai Caikang Optical Instrument Co., LTD., China). The migrated cells were counted by in 5 or more random fields of view.

### FISH

Fluorescence *in situ* hybridization was conducted using the Fam-labeled probe Lnc-PDZD7-3 (probe sequence CTC​GCG​TGA​AGT​TCC​CTT​CT), with U6 and GAPDH serving as the reference genes for nucleus and cytoplasm, respectively. The fluorescence *in situ* hybridization kit (Ribobio, Guangzhou, China) was employed to assess the subcellular localization of Lnc-PDZD7-3. Finally, the nuclei of Lnc-PDZD7-3 were stained with DAPI and examined with a fluorescence microscope.

### Statistical analysis

All experiments were conducted at least three times and data were expressed as mean ± standard deviation. The data were statistically analyzed using SPSS Vision 19.0 (SPSS, Chicago, Illinois, United States). The data of TCGA were normalized by means of the collaborator method, the dispersion of 57 pairs of samples was estimated, and then the differentially expressed genes were found by the general linear model. Differences in expression level of Lnc-PDZD7-3 in different clinical data and different pathological features were compared using Mann-Whitney U test. Two groups were compared with the Student t test. One-way ANOVA followed by Dunnett test was used to assess differences between more than two groups. p < 0.05 indicates statistical significance of the data.

## Results

### Lnc-PDZD7-3 is upregulated in LUAD and predicts poor prognosis

**FIGURE 1:**
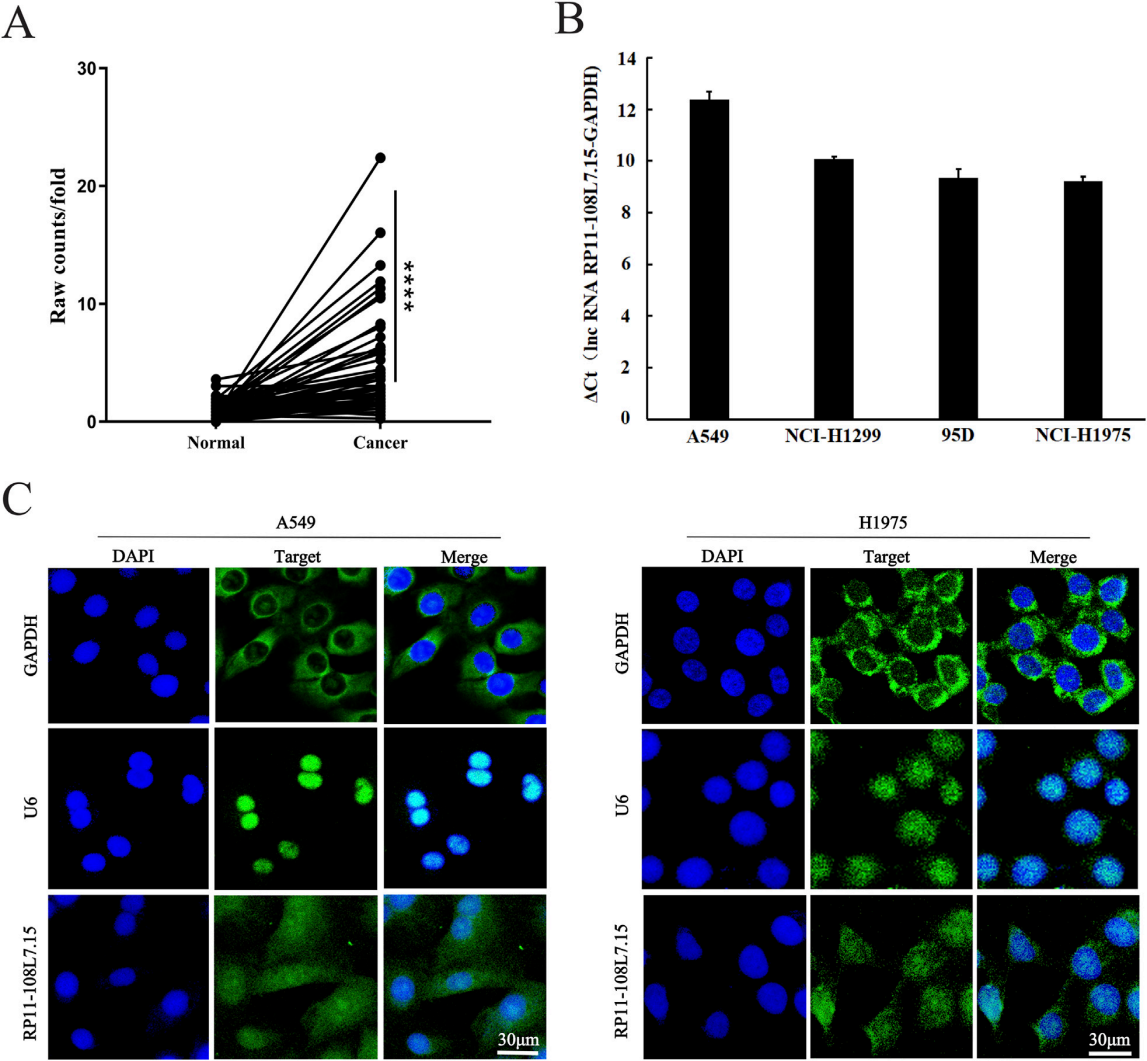
Lnc-PDZD7-3 is upregulated in LUAD. **(A)** Relative expression levels of Lnc-PDZD7-3 in lung adenocarcinoma tissues compared with corresponding non-tumor tissues. **(B)** In all four LUAD cell lines (A549, H1299, 95D and H1975), the expression level of Lnc-PDZD7-3 was medium in A549 and high in the other three cell types **(C)** Distribution of Lnc-PDZD7-3 in A549 andH1975 cells (Scale bar, 30 μm). A549 andH1975cells were subjected to fluorescent *in situ* hybridization (FISH) analysis using probes against Lnc-PDZD7-3.

According to TCGA database, in 57 pairs of cancer and adjacent tissues, the expression of Lnc-PDZD7-3 in LUAD tissues was enhanced relative to the normal lung tissues (Figure 1A). More importantly, Lnc-PDZD7-3 expression profile varied in cancer tissues of patients with different M metastasis (p < 0.05), suggesting that the expression level of Lnc-PDZD7-3 could be used as an indicator for the clinical diagnosis of distal metastasis (Table 2). The expression of Lnc-PDZD7-3 was positively correlated with the level of M metastasis, implying that the levels of this gene were higher in patients with distal metastasis, and patients with M metastasis predicted poor outcome (Table 2). In all four LUAD cell lines (A549, H1299, 95D and H1975), the expression level of Lnc-PDZD7-3 was medium in A549 and high in the other three cell types (Figure 1B). The A549 and H1975 cells were chosen as the target cells for RNA interference to detect the knockdown efficiency of Lnc-PDZD7-3 and subsequent tests. The subcellular distribution assay suggested that Lnc-PDZD7-3 was located predominantly in the cell nuclear and cytoplasm as a punctate pattern of LUAD cells (Figure 1C).

**TABLE 2:** The results between the expression level of Lnc-PDZD7-3 and the clinical diagnosis of distal metastasis.

Characteristics		LncPDZD7-3		Overall	P value
		Low	High		
T stage	T1	91	77	168	0.736
	T2	125	151	276	
	T3	27	20	47	
	T4	12	7	19	
Overall		255	255	510	
N stage	N0	164	166	330	0.998
	N1/2/3	85	86	171	
Overall		249	252	501	
M stage	M0	178	166	344	0.022
	M1	7	18	25	
Overall		185	184	369	
TNM	StageI	139	135	274	0.272
	StageII	67	54	121	
	StageIII	38	46	84	
	Stage IV	8	18	26	
Overall		252	253	505	

### Knockdown of Lnc-PDZD7-3 downregulates the proliferation of LUAD cells

**FIGURE 2:**
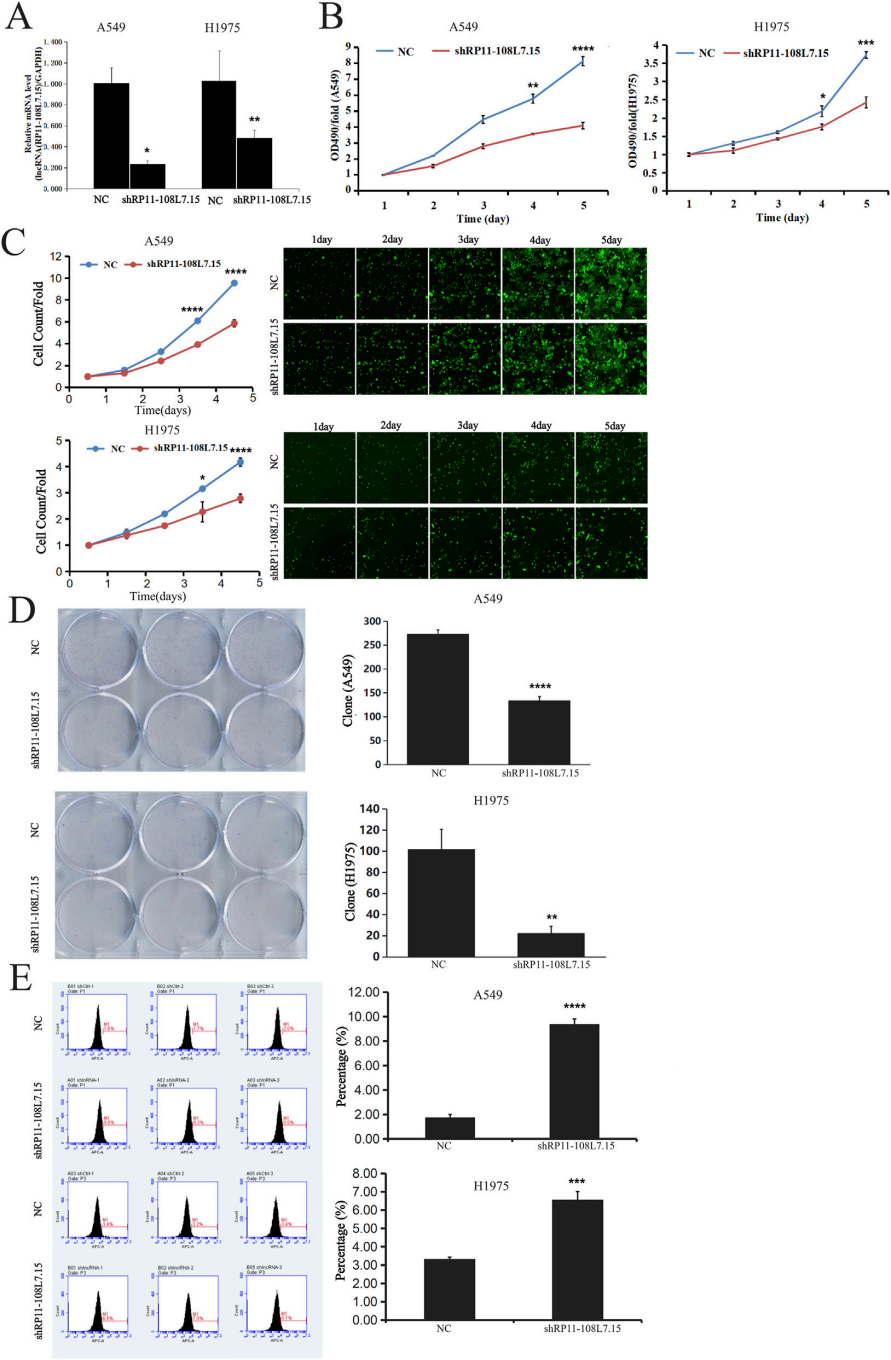
Knockdown of Lnc-PDZD7-3 reduces proliferation and promotes apoptosis in LUAD cells. **(A)** Analysis of PCR results showed that shRNA lentivirus infection downregulated mRNA expression of Lnc-PDZD7-3 gene in A549 and H1975 cells (p < 0.05), and the knockdown efficiency reached 76.3% and 51.5%, respectively. **(B)** D490 reflected the number of viable cells, and we found that the transfection of shLnc-PDZD7-3 significantly impaired the viability of the A549 and H1975 cell lines. **(C)** The Celigo cell count showed that the proliferation of multiple of A549 and H1975 cells were lower after Lnc-PDZD7-3 expression was inhibited for 5 consecutive days relative to the control group. **(D)** The results showed that the transfection of Lnc-PDZD7-3 significantly impaired the viability of the A549 and H1975 cell lines. **(E)** The results showed that the transfection of Lnc-PDZD7-3 significantly increased apoptosis of A549 and H1299 cells.

Based on the high expression of Lnc-PDZD7-3 in LUAD tissues, we hypothesized that Lnc-PDZD7-3 can accelerate LUAD cells proliferation. Analysis of PCR results showed that shRNA lentivirus infection downregulated mRNA expression of Lnc-PDZD7-3 gene in A549 and H1975 cells (p < 0.05), and the knockdown efficiency reached 76.3% and 51.5%, respectively (Figure 2A). Meanwhile, D490 reflected the number of viable cells, and we found that the transfection of shLnc-PDZD7-3 significantly impaired the viability of the above two LUAD cells (Figure 2B, P < 0.05). As shown in Figure 2C, the results of the Celigo cell count showed that the proliferation of multiple of A549 and H1975 cells were lower after Lnc-PDZD7-3 expression was inhibited for 5 consecutive days relative to the control group. Furthermore, transfection of shLnc-PDZD7-3 into A549 and H1975 cells resulted in a significant decrease in clonogenesis and an increase in apoptosis (Figures 2D,E). This is thought to be due to knockdown of Lnc-PDZD7-3 which attenuates its promotion of proliferation in LUAD cells.

### Lnc-PDZD7-3 knockdown downregulated the migration of LUAD cells

**FIGURE 3:**
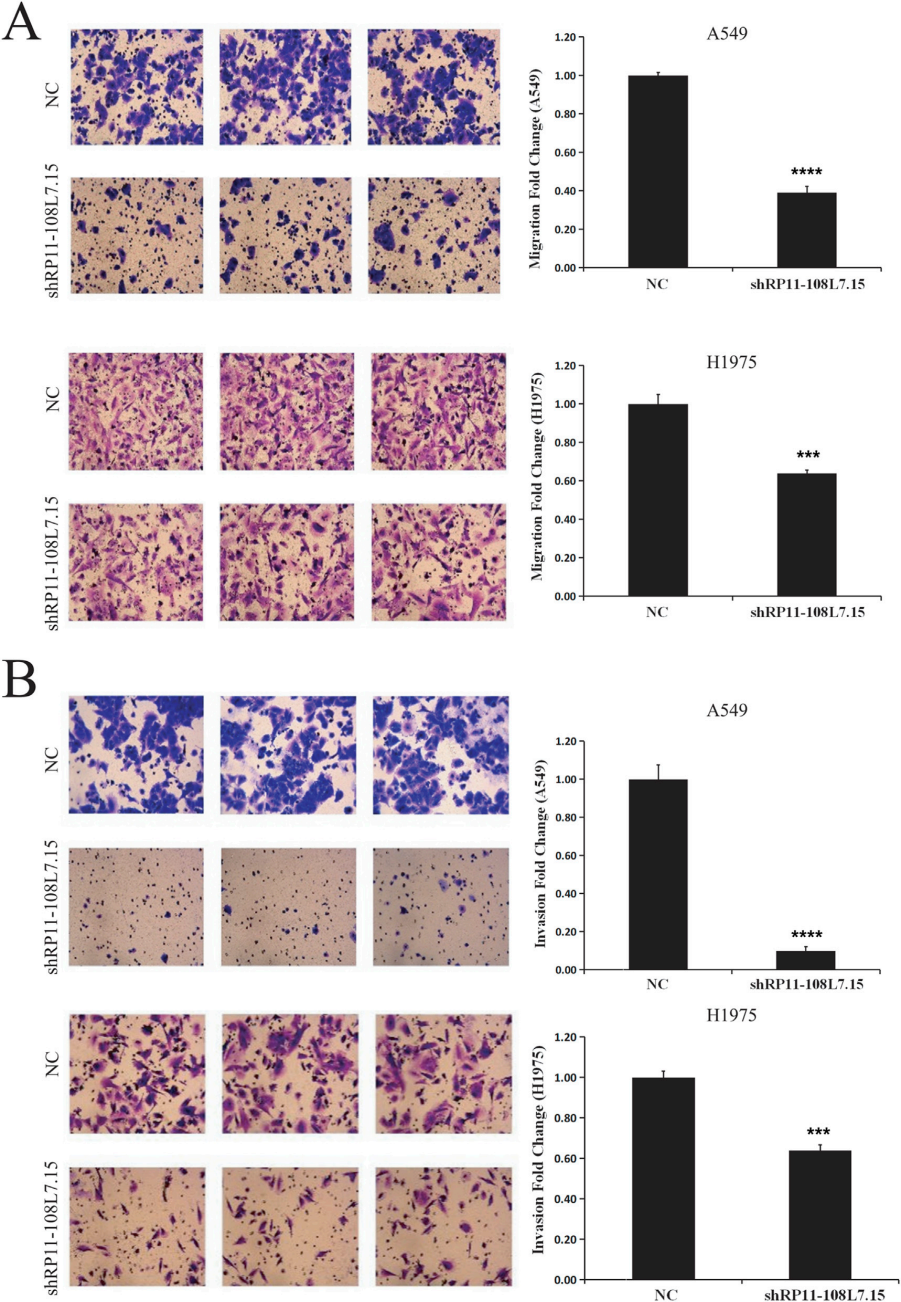
Knockdown of Lnc-PDZD7-3 reduces the migration and invasion abilities of LUAD cells. **(A)** By examining the effect of Lnc-PDZD7-3 on the metastasis ability of LUAD cells, we found that compared with the control group, the migration of A549 and H1299 cells in the shlncRNA group was inhibited. **(B)** Invasion from extracellular matrix is an important step in tumor cell metastasis, and invasion chamber assay showed that knockdown of Lnc-PDZD7-3 significantly inhibited the invasion ability of A549 and H1975 cells.

LUAD has a high metastatic potential in the early stage of cancer, which reduces the effect of early tumor resection and treatment, and the long-term outcome of patients is not ideal. By examining the effect of Lnc-PDZD7-3 on the metastasis ability of LUAD cells, it was observed that the migration of A549 and H1299 cells in the shlncRNA group was lower relative to the control group (Figure 3A, P < 0.05). Invasion from extracellular matrix is an important step in tumor cell metastasis, and invasion chamber assay showed that knockdown of Lnc-PDZD7-3 resulted in marked inhibition of the invasion ability of A549 and H1975 cells (Figure 3B, P < 0.05). Therefore, we conclude that Lnc-PDZD7-3 influences the migration of LUAD, which may enhance the invasion ability of LUAD cells to invade from the extracellular matrix into normal tissues and thus promote the metastasis of tumor cells.

### Lnc-PDZD7-3 promotes the progression of LUAD by activating FN1 gene

**FIGURE 4:**
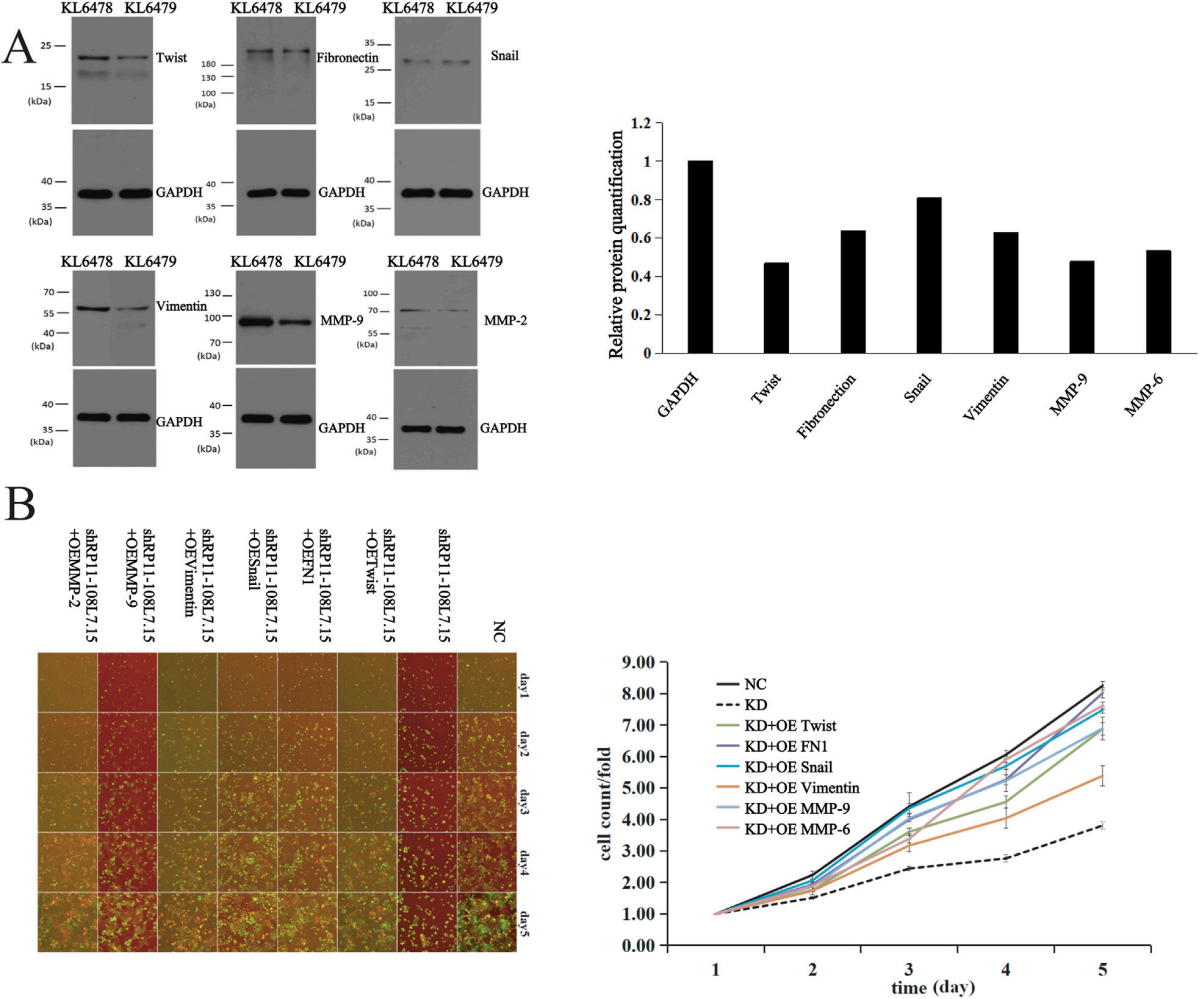
Lnc-PDZD7-3 promotes the development of LUAD by regulating FN1. **(A)** Wesern blot analysis showed that knockdown of LncPDZD7-3 downregulated the expression of all six proteins, especially fibronectin. **(B)** The results showed that compared with NC group, the proliferation of Knockdown group was significantly slowed down, while the proliferation trend of OE group was recovered after gene overexpression, and the FN1 gene recovered most obviously.

To explore the role of Lnc-PDZD7-3 in LUAD development, six genes associated with tumor initiation and metastasis via the classic signaling pathway were analyzed: Twist1, fibronectin, q, vimentin, MMP - 9, MMP - 2. It was found that Lnc-PDZD7-3 knockdown down-regulated the expression of all six proteins, especially fibronectin (Figure 4A). A549 cells were selected as transfected cells, and the proliferation of genes encoding these six proteins was screened. The results indicate that the proliferation of Knockdown group was lower relative to the NC group, while the proliferation trend of OE group was recovered after gene overexpression, and the FN1 gene recovered most obviously (Figure 4B). Fibronectin is produced by FN1 transcription and translation, and its role is to activate the regenerative and migratory ability of target cells. Cell viability test showed that compared with NC group, the proliferation ability of A549 cells in Knockdown group was significantly decreased, while the proliferation ability of A549 cells in OE (FN1) group was significantly restored. At the same time, transwell experiment also proved that compared with the Knockdown group, the metastasis rate of A549 cells was higher in OE (FN1) group. These results further demonstrated that Lnc-PDZD7-3 could positively regulate FN1 gene to promote the expression of Fibronectin, thereby enhancing the migration and proliferation ability of LUAD cells. FISH experiments revealed that Lnc-PDZD7-3 was present in both the nucleus and cytosol, indicating that this regulatory mechanism was only one but not the only one.

## Discussion

The involvement of lncRNA in the development of drug resistance, invasion, migration, and proliferation of cancer cells has been reported in many studies (Sun et al., 2016; Zhang et al., 2016; Shao et al., 2016). Notably, LncRNA can regulate histone modification, chromatin remodeling, and inhibit transcription factors in NSCLC. It has also been demonstrated that lncRNA can bind proteins or function as the precursor molecules of small molecule RNA (Ricciuti et al., 2016; Kunz et al., 2016). Other researchers have uncovered that lncRNA can influence multiple signaling pathways to modulate the progression of NSCLC. Here, we explored the role of a previously unreported long noncoding RNA: Lnc-PDZD7-3 in LUAD. This study shows that the expression of Lnc-PDZD7-3 in lung adenocarcinoma tissues is higher than that in corresponding non-cancer tissues, and the higher expression of Lnc-PDZD7-3 is associated with distant metastasis in lung adenocarcinoma patients, which means that the difficulty of treatment is increased, indicating poor survival and prognosis. We found that silencing Lnc-PDZD7-3 inhibited the proliferation and viability of LUAD cells and accelerated apoptosis *in vitro*, reflecting the promoting effect of Lnc-PDZD7-3 on the proliferation of LUAD cells. Western blot analysis found that in LUAD cells, silencing Lnc-PDZD7-3 can down-regulate the expression of various related proteins, including Twist1, fibronectin, snail, vimentin, MMP - 9, MMP - 2, may be involved in the development of LUAD. FN1, a glycoprotein, has major roles in cell growth, differentiation, migration, and adhesion, and is vital for embryonic development and wound-healing (Sung et al., 2011). Degradation or alteration of FN1 expression has been associated with cancer progression, such as in squamous cell carcinoma, nasopharyngeal carcinoma, ovarian cancer, and renal cancer (Sung et al., 2011; Xiao et al., 2018; Takayasu et al., 2001). Previous studies have proved that the extracellular matrix glycoprotein, fibronectin, regulates cell differentiation, migration and adhesion. Strategies targeting FN are potential treatments for cancer (Abdel-Ghany et al., 1998; Janker et al., 2019; Berndorff et al., 2005). Therefore, we concluded that FN1 may be a key regulatory point of Lnc-PDZD7-3 in promoting the growth and metastasis of LUAD cells. When downstream genes were overexpressed, the proliferation capacity of LUAD was significantly restored compared with Lnc-PDZD7-3 knockdown group, and the most obvious recovery was found in FN1 group. Evidence from prior studies show that FN1 enhances glioma development by interacting with integrin b, and activating MMP2/MMP9 to accelerate the invasion and metastasis of cancer cells (Song et al., 2021). And, Tan et al. found that HOXD11 activated the transcription of FN1 to decompose the extracellular matrix and to promote in penile squamous cell carcinoma metastasis via FN1/MMP2/MMP9 pathways (Tan et al., 2022). This also explains that the response of LUAD increment ability in FN1 group is much higher than that in MMP2 and MMP9 groups. MMT and Tanswell assay results demonstrated that the viability and metastasis ability of LUAD cells in FN1 overexpression group were markedly increased than those in shLnc-PDZD7-3 group, which further confirmed that Lnc-PDZD7-3 up-regulated the expression of fibronectin by positively regulating FN1, which enhanced that metastasis and proliferation of LUAD cells. Based on the significant role of Lnc-PDZD7-3 in cancer, modulating the expression level of Lnc-PDZD7-3 for tumor treatment is a very promising approach. Therefore, we can consider using methods such as silencing LncRNA or small molecule inhibitors to achieve the therapeutic goal. Although the application of LncRNA in treatment still faces many challenges, it has also brought new perspectives and breakthroughs to research.

This study has some limitations. FISH assays revealed that Lnc-PDZD7-3 was present in both the nucleus and cytosol, indicating that Lnc-PDZD7-3 may synergistically promote the development of LUAD through various ways. This study demonstrated the signaling transduction role of the Lnc-PDZD7-3 -FN1-fibronetin pathway axis in the progression of LUAD. However, how Lnc-PDZD7-3 regulates the transmission process of FN1 should involve other genes and regulatory patterns. Additionally, based on the role of Lnc-PDZD7-3 in LUAD, we conducted *in vitro* experiments, but conducting *in vivo* experiments would better clarify its function. Therefore, the clinical application of Lnc-PDZD7-3 requires more experiments and further research, which is also the direction of our next research.

## Conclusion

In summary, our work proved that Lnc-PDZD7-3 promoted the LUAD progression, and also revealed the signal transduction pathway of Lnc-PDZD7-3 -FN1-fibronetin in the progress of LUAD. This study indicates that Lnc-PDZD7-3 may be a new diagnostic marker of LUAD and a potential therapeutic target for LUAD. However, more evidence is needed in the future to enhance the clinical value of drugs targeting Lnc-PDZD7-3.

## Data Availability

The original contributions presented in the study are publicly available. This data can be found here: https://doi.org/10.6084/m9.figshare.30354103.v1.

## Abbreviations

LUAD, lung adenocarcinoma; NSCLC, non-small cell lung cancer; TCGA, the Cancer Genome Atlas; PCR, Polymerase Chain Reaction; NC, Control group; OE, Overexpression group.
